# Enhanced Neuroprotective Effects of *Panax ginseng* G115^®^ and *Ginkgo biloba* GK501^®^ Combinations In Vitro Models of Excitotoxicity

**DOI:** 10.3390/ijms20235872

**Published:** 2019-11-22

**Authors:** Elisa Landucci, Domenico E. Pellegrini-Giampietro, Anna Rita Bilia, Maria Camilla Bergonzi

**Affiliations:** 1 Department of Health Sciences, Section of Clinical Pharmacology and Oncology, University of Florence, Viale Pieraccini 6, 50139 Florence, Italy; domenico.pellegrini@unifi.it; 2 Department of Chemistry, University of Florence, Via Ugo Schiff 6, 50019 Sesto Fiorentino, Florence, Italy; ar.bilia@unifi.it (A.R.B.); mc.bergonzi@unifi.it (M.C.B.)

**Keywords:** excitotoxicity, neuroprotection, *Ginkgo biloba* GK501^®^, *Panax ginseng* G115^®^, organotypic hippocampal slices, cortical cells

## Abstract

Neurological-related disorders are seen as an increasingly important aspect of welfare. While conventional medicine is still the mainstay for the treatment of these diseases, it is becoming apparent that patients are also seeking more natural and preventative interventions. *Panax ginseng* G115^®^ and *Ginkgo biloba* GK501^®^ extracts alone or in combination were used in two in vitro experimental models of primary cultures exposed to excitotoxicity: rat organotypic hippocampal slices exposed to either 5 µM kainic acid or 10 µM *N*-Methyl-d-aspartate for 24 hours, and mixed cortical cells exposed to 300 µM NMDA for 10 min. Cell death in the *Cornu Ammonis* areas CA3 or CA1 subregions of slices was quantified by measuring propidium iodide fluorescence, whereas in cortical cells, it was assessed by measuring the amount of lactate dehydrogenase. In slices, treatment with extracts alone or in combination significantly attenuated CA3 and CA1 damage induced by exposure to kainic acid or NMDA, respectively. A similar neuroprotective effect was observed in cortical cells exposed to NMDA. Analysis of cell signaling pathways found that the two extracts induced an increase of the phosphorylation and they reversed the decrease of phosphorylation of ERK1/2 and Akt induced by kainic acid and NMDA in organotypic hippocampal slices. These results suggest that *P. ginseng* G115^®^ and *G. biloba* GK501^®^ extracts may mediate their effects by activating phosphorylation of ERK1/2 and Akt signaling pathways, protecting against excitotoxicity-induced damage in in vitro models.

## 1. Introduction

Glutamate is considered to be an important excitatory neurotransmitter that mediates it effects by binding to and activating ionotropic and metabotropic glutamate receptors in the brain [1]. Both in vitro and in vivo studies have demonstrated that over activation of ionotropic glutamate receptors (such as α-amino-3-hydroxy-5-methyl-4-isoxazolepropionic acid (AMPA), *N*-methyl-d-aspartate (NMDA), and kainate receptors) through exposure to high levels of glutamate can lead to excitotoxicity. It has been hypothesized that chronic excitotoxicity contributes to the onset of a number of neurodegenerative diseases such as amyotrophic lateral sclerosis (ALS) and Alzheimer’s disease [1]. The NMDA and the two “non-NMDA” kainate and AMPA receptors have subtly different functions within the brain. The AMPA receptors are predominantly responsible for mediating the influx of sodium while the kainate receptors regulate the influx of both sodium and potassium ions. The NMDA receptors have the greatest conductivity by allowing the influx of positive ions. While both NMDA and kainic acid are physiological ligands for these receptors, over-activation can result in excitotoxicity due to an increased influx into the cell of calcium ions (Ca^2+^). Increase in the cellular concentration of Ca^2+^ activates enzymatic signaling cascades that are integral to the regulation of numerous cellular functions including the oxidative stress response, inflammation, mitochondrial impairment, and caspase activation. The dysregulation of these pathways ultimately leads to the death of neuronal cells. In recent decades, the interest in herbal medicines as a therapeutic resource in disease prevention, health promotion, and recovery has grown in many countries. There is some evidence demonstrating the use of botanicals as useful modulators of Brain-derived neurotrophic factor (BDNF) in central nervous system (CNS) diseases [2]. Further preclinical and clinical studies are needed to support the safe use of herbal medicines as prophylactic and/or therapeutic strategies for the treatment of neurodegenerative diseases [2].

Over the last decade there has been increasing evidence to support the therapeutic benefits of *Panax ginseng* and *Ginkgo biloba* and their components in neurodegenerative brain disease [3,4]. The beneficial effects observed have been attributed predominantly, but not exclusively, to the immunomodulatory and antioxidative properties of the herbal medicines. The pharmacological effects of *P. ginseng* are due largely to the action of ginsenosides, which are considered to be the major active components. However, other bioactive ingredients of *P. ginseng,* such as the phytosterols, sesquiterpene, flavonoids, polyacetylese, alkaloids, and phenolic compounds, are involved in the important role of eliciting the beneficial effects of the ginsenosides [5,6,7,8]. Water extract of *P. ginseng* has been demonstrated to have a protecting effect against 1-methyl-4-phenylpyridinium-iodide (MPP+)-induced apoptosis in in vitro models of Parkinson’s disease [9]. Other studies have demonstrated that ginsenoside Rb1 can protect dopaminergic neurons, SH-SY5Y cells, and PC12 cells from 6-OHDA- or MPP+-induced toxicity [10,11,12]. Ginsenoside Rd has been demonstrated in male ischemic rat models to increase extracellular glutamate clearance by the upregulation of GLT-1 expression, mediated by the activation of PI3K/AKT and ERK1/2 signaling pathways [13]. Further to this, Ginsenoside Rd has been shown to decrease levels of apoptotic proteins such as PARP1 and Bax, via adenylate cyclase-associated protein 1 (CAP1) regulation in an in vitro model of Alzheimer’s disease [14]. Ginsenoside Rg1 reduced the amyloid β-stimulated expression of Toll-like receptors and TNF-α in a NG108-15 neuroglia cell line. *P. ginseng* extracts showed neuroprotective effects by ameliorating the advanced glycation end-product-induced memory impairment and reducing the pathophysiological changes through down regulation of the RAGE/NF-kB pathway [15]. Furthermore, in Alzheimer-like rat models, ginsenoside reduced the d-galactose- and aluminum chloride (AlCl3)-induced spatial memory impairment through restoration of neurotransmitter levels, tau phosphorylation, and amyloid β formation [16]. In an in vitro model of Huntington’s disease, ginsenosides protected striatal neurons in an Huntington’s disease (HD) mouse model from glutamate toxicity [17].

Research conducted with the *G. biloba* Egb 761^®^ extract (containing 49% total flavones; 28.7% glycosides; 11.6% gingkolides (sum of A, B, C, and bilobalide); and 3.3% gingkolide A) in human astrocytes demonstrated reduced neuroinflammation by blocking the generation of pro-inflammatory cytokines and oxygen-glucose deprivation (OGD)-induced signal transducer and activator of transcription (STAT3) activation [18]. The same authors observed that *G. biloba* Egb761^®^ was able to attenuate cerebral infarction and neuronal apoptosis and reduce neurological deficiencies in cerebral ischemic rats [18]. The extract inhibited the Aβ induced activation of NF-κB and MAPK pathways in the neuroblastoma cell line N2a, thereby protecting the neuronal cells from Aβ toxicity [19]. Kim and colleagues observed that pretreatment with daily administration of Egb761^®^ extract induced a neuroprotective effect on 6-hydroxydopamine (6-OHDA)-induced neurotoxicity in the rat brain [20]. The neuroprotective effects of *G. biloba* correlated to the regulation of the content of copper in the brain, as observed in animal models of Parkinson’s disease [21]. In vitro studies with PC12 neuronal cells investigating Aβ (1–42) treatment (aggregated and soluble form) showed that *G. biloba* extracts have the potential to prevent Aβ-induced reactive oxygen species (ROS) production, cytotoxicity, glucose uptake, and apoptosis as well as the development of Aβ-derived diffusible neurotoxic ligands. These neurotoxic ligands have been implicated in mediating the neurotoxic effect of Aβ [22]. In C. elegans, *G. biloba* Egb761^®^ alleviates Aβ-induced pathological behavior, inhibits Aβ oligomerization and deposits (not by reducing oxidative stress), and attenuates both the basal and Aβ-induced levels of H_2_O_2_-related reactive oxygen species in Alzheimer’s disease models of neurodegeneration [23,24]. A study conducted by Liu et al. using a transgenic mouse model investigated the anti-inflammatory activity and underlying molecular pathways impacted by treatment with *G. biloba* extract in the context of Alzheimer’s disease. The results showed inhibition of neuroinflammation, reduction of cognitive deficit and synaptic impairment, and enhanced autophagy, as well as the prevention of Aβ-induced microglial inflammatory activation [25]. In a recent systematic review, Reay and colleagues investigated both the psychological and physiological impact of the combination of *G. biloba* GK501^®^ and *P. ginseng* G115^®^ (Gincosan^®^) in human subjects. In particular, the most interesting results were the observed benefits of treatment on both the circulatory and cardiovascular systems as well as the improvement in the “secondary memory” performance in the total patient population. The conclusion of that review was that the Gincosan^®^ treatment can improve the physiological and cognitive functions in humans [26].

In the present study, in vitro models of excitotoxicity were used to examine the neuroprotective effects of *P. ginseng* G115^®^ and *G. biloba* GK501^®^ extracts. The extracts were tested alone and in combination (mix), maintaining the same ratio as the commercial product Gincosan^®^.

## 2. Results

### 2.1. Safety and Tolerability of Native P. Ginseng and G. Biloba GK501^®^ Extracts Alone or as a Mix in Rat Organotypic Hippocampal Slices

**Figure 1:**
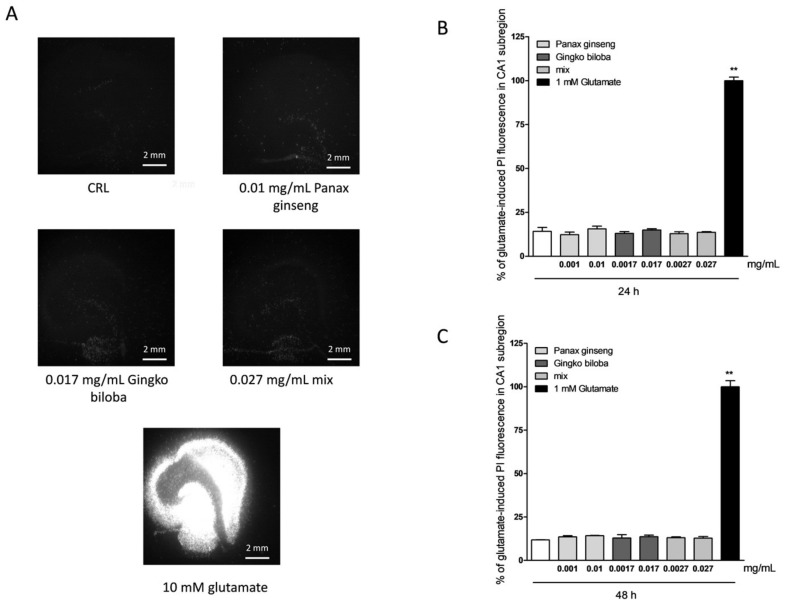
Effects of native *P. ginseng* and *G. biloba* GK501^®^ extracts alone or in combination in organotypic hippocampal slice cultures in basal conditions. (**A**) Top left:Hhippocampal slice under normal conditions (background PI fluorescence). Hippocampal slices incubated with 0.01mg/mL native *P. ginseng* (top right), 0.017 mg/mL *G. biloba* GK501^®^ (bottom left) and 0.027 mg/mL mix extracts (bottom right) for 24 h under normoxic conditions. Bottom center: Slice exposed to 10 mM glutamate for 24 h, displaying intense PI labeling in all sub-regions. Quantitative analysis at 24 h (**B**) and 48 h (**C**) incubation with extracts. Bars represent the mean ± SEM of at least four experiments run in quadruplicate. ** *p* < 0.01 vs. control (CRL) (ANOVA + Dunnet’s test).

To investigate the safety and tolerability of native *P. ginseng* and *G. biloba* GK501^®^, rat organotypic hippocampal slices were incubated with increasing concentrations of extract alone or as mix and the *Cornu Ammonis* areas CA1 and CA3 regions were evaluated for damage using PI fluorescence (Figure 1A). Quantitative analysis of hippocampal slices exposed for 24 h (Figure 1B) or 48 h (Figure 1C) to native *P. ginseng* (0.001–0.01 mg/mL), *G. biloba* GK501^®^ (0.0017–0.017 mg/mL), and/or a combination (mix 0.0027–0.027 mg/mL) showed that these drugs did not induce injury in either the CA1 or CA3 region in this model, demonstrating that they were well-tolerated as compared to the exposure with 10 mM glutamate (24 h), which has been demonstrated previously to result in maximal injury [27], and was used as a positive control (Figure 1A).

### 2.2. Neuroprotective Effects of Native P. Ginseng and G. Biloba GK501^®^ Extracts Alone or as a Mix in Rat Organotypic Hippocampal Slices Exposed to Kainic Acid or NMDA-Induced Neurotoxicity

**Figure 2:**
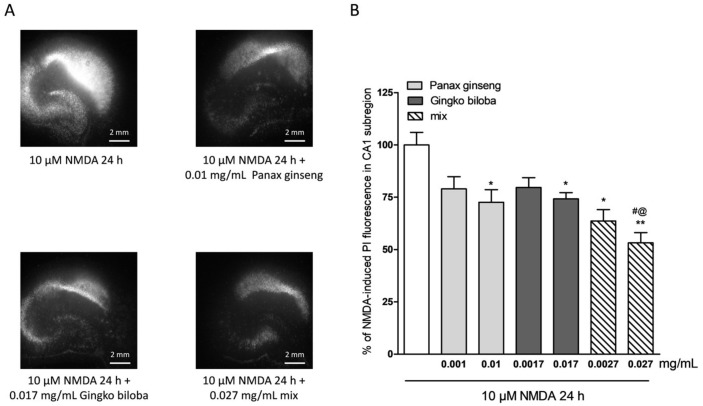
Effects of native *P. ginseng* and *G. biloba* GK501^®^ extracts alone or in combination in slice cultures exposed to NMDA. (**A**) Hippocampal slices exposed to 10 µM NMDA for 24 h displaying intense PI labeling in the CA1 subregion. *P. ginseng*, *G. biloba* and mix extracts reduced the PI labeling induced by 10 µM NMDA for 24 h in the CA1 area of slices. (**B**) Quantitative analysis of the effects of native *P. ginseng* and *G. biloba* GK501^®^ extracts alone during the 24 h NMDA period, reduced NMDA injury in CA1. Bars represent the mean ± SEM of at least four experiments run in quadruplicate. (* *p* < 0.05 and ** *p* < 0.01 vs. NMDA # *p* < 0.05 vs. 0.01; @ *p* < 0.05 vs. 0.017) (ANOVA + Tukey’s *w*-test).

**Figure 3:**
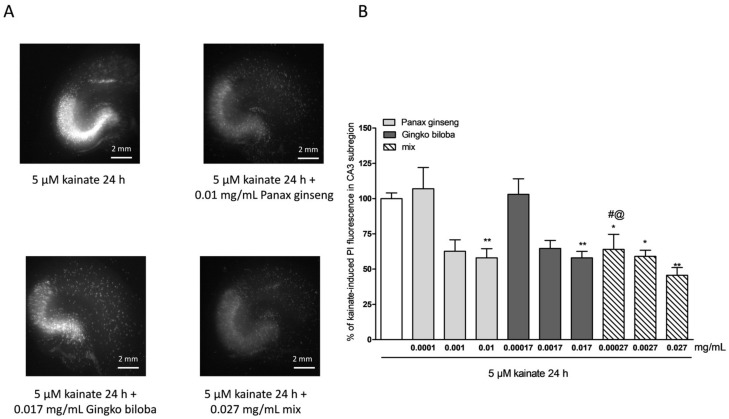
Effects of single native *P. ginseng* and *G. biloba* GK501^®^ extracts or a mix in slice cultures exposed to kainic acid. (**A**) Slice exposed to 5 µM kainate for 24 h displaying intense PI labeling in the CA3 subregion. *P. ginseng*, *G. biloba* and mix extracts reduced PI labeling induced by 5 µM kainic acid for 24 h in the CA3 area of slices. (**B**) Quantitative analysis of the effects of native *P. ginseng* and *G. biloba* GK501^®^ extracts alone during the 24 h kainate period, reduced kainate injury in CA3. Bars represent the mean ± SEM of at least four experiments run in quadruplicate. * *p* < 0.05 and ** *p* < 0.01 vs. kainite # *p* < 0.05 vs. 0.0001; @ *p* < 0.05 vs. 0.00017 (ANOVA + Tukey’s *w*-test in (B)).

To investigate the neuroprotective effects of the extracts, rat organotypic hippocampal slices were incubated with 10 µM NMDA or 5 µM of kainic acid for 24 h to induce selective injury to the CA1 and CA3 areas, respectively (Figure 2A and Figure 3A). Native *P. ginseng* and *G. biloba* GK501^®^ extracts alone and as a mix reduced the CA1 NMDA-induced injury, in particular, both extracts alone had neuroprotective effects that became significant at the high doses, 0.01 mg/mL for the native *P. ginseng* and 0.017 mg/mL for *G. biloba* GK501^®^ (Figure 2A,B). The mix had an additive protective effect against the NMDA induced damage (* *p* < 0.05, ** *p* < 0.01 vs. NMDA; # *p* < 0.05 vs. 0.01; @ *p* < 0.05 vs. 0.017; Figure 2B). Similarly, when slices were exposed to extracts during kainic acid treatment, we observed that the neuronal damage in the CA3 region was attenuated (Figure 3A). Quantitative analysis showed that kainic acid-induced injury was significantly reduced at high doses of the single extracts alone, whereas the mix induced a notably neuroprotective effect (Figure 3B) (* *p* < 0.05, ** *p* < 0.01 vs. kainic acid; # *p* < 0.05 vs. 0.0001).

### 2.3. Safety and Tolerability of Native P. Ginseng and G. Biloba GK501^®^ Extracts Alone or as a Mix in Cortical Mice Cells

**Figure 4:**
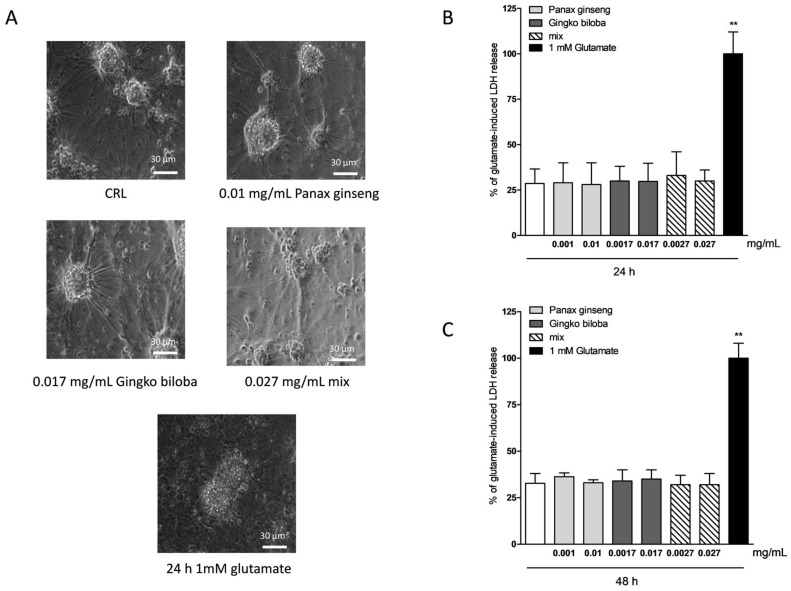
Effects of native *P. ginseng* and *G. biloba* GK501^®^ extracts alone or as a mix in murine mixed cortical cell cultures in basal conditions. Cells were exposed to extracts, and 24–48 h later neuronal injury was assessed by phase-contrast microscopy or by measuring the release of lactate dehydrogenase (LDH). (**A**) Cortical culture in normal conditions and treated with increasing concentrations of extracts for 24–48 h displaying intact morphology. Cells exposed to 1 mM glutamate for 24 h, displaying ample death of neuronal bodies. (**B**,**C**) Quantitative analysis of extracts in mixed cortical cells. Data are expressed as percentage of glutamate-induced LDH release represented by the mean ± SEM of at least three experiments performed in triplicate. ** *p* < 0.01 vs. CRL (ANOVA + Dunnet’s test).

To investigate the safety and tolerability of the extracts in other areas of the brain, murine cortical cells were incubated with increasing concentrations of extract alone or in combination, and cell damage was assessed by measuring the release of lactate dehydrogenase (LDH) (Figure 1A). Cortical cells exposed for 24 h to native *P. ginseng* (0.01 mg/mL), *G. biloba* (GK501^®^) (0.017 mg/mL), and mix (0.027 mg/mL) showed no apparent signs of injury (Figure 4A), whereas the maximal damage was achieved in this system by exposing cells to 1 mM of glutamate for 24 h (Figure 4A) [26]. Quantitative analysis showed that these extracts were well tolerated when present in the incubation medium for 24 h (Figure 4B) and for 48 h (Figure 4C) at the concentrations of native *P. ginseng* (0.001–0.01 mg/mL), *G. biloba* GK501^®^ (0.0017–0.017 mg/mL), and/or a combination of extracts (mix 0.0027–0.027 mg/mL) (Figure 4B; ** *p* < 0.01 vs. control (CRL)).

### 2.4. Neuroprotective Effects of Native P. Ginseng and G. Biloba GK501^®^ Extracts Alone or as a Mix in Cortical Mice Cell Cultures Exposed to NMDA-Induced Neurotoxicity

**Figure 5:**
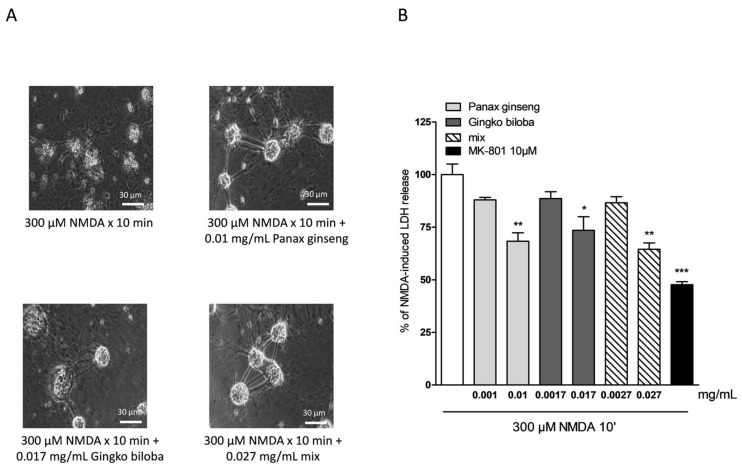
Effects of native *P. ginseng* and *G. biloba* GK501^®^ extracts alone or as a mix in murine mixed cortical cell cultures treated with 300 µM NMDA for 10 min. Cultures were exposed to 300 µM NMDA for 10 min, and 24 h later neuronal injury was assessed. The extracts were present in the incubation medium for 10 min of NMDA treatment and maintained for a 24 h recovery period. (**A**) Cell treated with NMDA, displaying extensive death of neuronal bodies. Neurons exposed to excitotoxicy and treated with extracts, displaying a reduction of NMDA injury. (**B**) Quantitative analysis of the effects of extracts alone or in combination in mixed cortical cells exposed to NMDA. Neuronal death was significantly reduced when cultures were treated with 0.01 mg/mL of native *P. ginseng* and 0.017 mg/mL of *G. biloba* GK501^®^ extracts and the maximal neuroprotective effect was obtained with the mix at 0.027 mg/mL. Data are expressed as percentage of NMDA-induced LDH release represented by the mean ± SEM of at least four experiments performed in triplicate. * *p* < 0.05, ** *p* < 0.01 and *** *p* < 0.001 vs. NMDA (ANOVA + Tukey’s *w*-test).

Native *P. ginseng* and *G. biloba* GK501^®^ extracts alone or as a mix elicited a dose-dependent neuroprotective effect that reached its maximal significance at 0.01 mg/mL for ginseng, 0.017 mg/mL for ginkgo, and 0.027 mg/mL for the mix (Figure 5A,B) when present in the incubation medium during the treatment with NMDA, and the subsequent 24 h recovery period. This protective effect was less than that obtained with the neuroprotective NMDA receptor blocker MK 801 at 10 µM (Figure 5B). In this model, no additive effect of the combination of the two extracts was observed, however, there was evidence of a prolonged protective response. 

**Figure 6:**
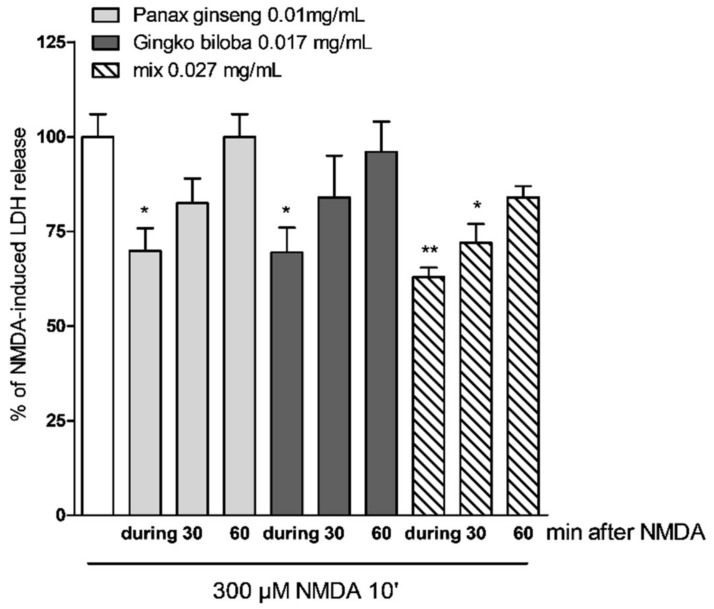
Effects of native *P. ginseng* and *G. biloba* GK501^®^ alone or as a mix at different time points in mixed cells exposed to excitotoxic insult. Quantitative analysis of the effects of extracts alone or in combination in mixed cortical cells at different time points after NMDA treatment. Cells treated with mix 30 min after the end of NMDA treatment displayed neuroprotective effects. Data are expressed as percentage of NMDA-induced LDH release represented by the mean ± SEM of at least four experiments performed in triplicate. * *p* < 0.05 and ** *p* < 0.01 vs. NMDA (ANOVA + Tukey’s *w*-test).

In cells treated with extracts at different time points after NMDA insult (0, 30, and 60 min), the protective effects of *G. biloba* GK501^®^ and native *P. ginseng* administered alone were lost, whereas with incubation with the mix, the neuroprotective effect could still be observed 60 min post NMDA insult. (Figure 6 * *p* < 0.05 and ** *p* < 0.01 vs. NMDA).

### 2.5. Effects of Native P. Ginseng and G. Biloba GK501^®^ Extracts Alone or as a Mix on MAP-Kinase/PI-3 Kinase Pathways

It has been reported that the activation of the MAP-kinase/PI-3 kinase pathways is associated with neuroprotective effects of natural or conventional drugs [9,25,26]. To determine if these pathways were involved in mediating the neuroprotective effects of native *P. ginseng* and *G. biloba* GK501^®^, we analyzed by Western blot the phosphorylation of Erk1/2 and Akt in organotypic slices and in mixed cortical cells.

**Figure 7:**
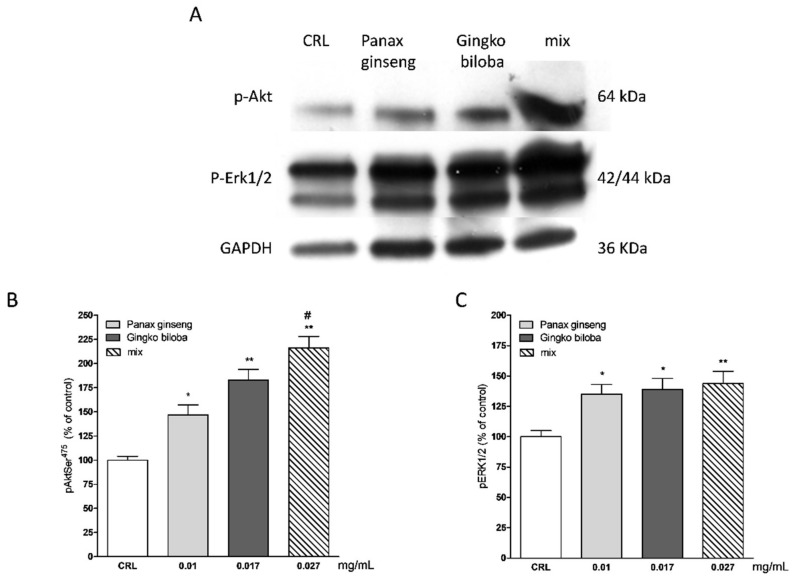
Effects of native *P. ginseng* and *G. biloba* GK501^®^ alone or in combination on MAP-kinase pathways in basal condition. (**A**) Illustrative blots using antibodies directed against phospho-ERK1/2 and phospho-Akt and GAPDH. Uncut original gels are available in the Supplementary File. (**B**,**C**) Quantitative analysis of immunoreactive bands, showing a significant increase in p-Akt and p-ERK1/2 protein levels induced by native *P. ginseng* (0.01 mg/mL) and *G. biloba* GK501^®^ (0.017 mg/mL) extracts alone or in combination (mix 0.027 mg/mL) exposure for 24 h. Data are expressed as percentage of phosphorylation in control untreated cultures (CRL). Bars represent the mean ± SEM of at least four experiments * *p* < 0.05, ** *p* < 0.01 vs. CRL; # *p* < 0.05 vs. 0.01. (ANOVA + Tukey’s *w*-test).

**Figure 8:**
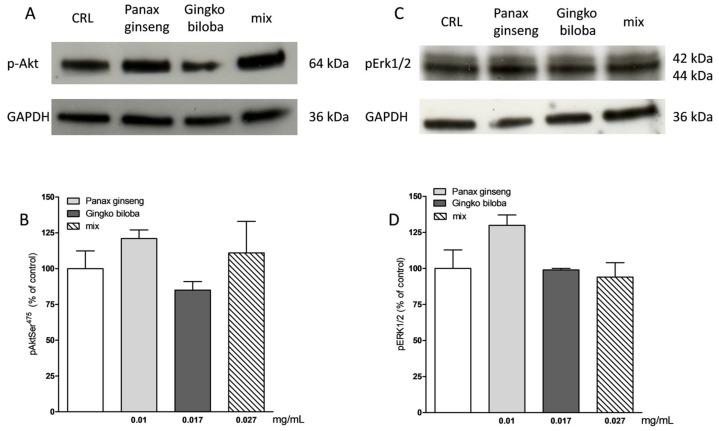
Effects of native *P. ginseng* and *G. biloba* GK501^®^ alone or in combination on MAP-kinase pathways in basal condition in mixed cortical cells. (**A**,**C**) Illustrative blots using antibodies directed against phospho-Akt and GAPDH and phospho-ERK1/2 and GAPDH. Uncut original gels are available in the Supplementary File. (**B**,**D**) Quantitative analysis of immunoreactive bands showing only a trend of increase in p-Akt and p-ERK1/2 protein levels induced by native *P. ginseng* (0.01 mg/mL) exposure for 24 h. Data are expressed as percentage of phosphorylation in control untreated cultures (CRL). Bars represent the mean ± SEM of at least four experiments.

Our results indicate that slices exposed for 24 h to 0.01 mg/mL of *G. biloba* GK501^®^, 0.017 mg/mL of *P. ginseng,* and 0.027 mg/mL of the mix showed significantly higher levels of p-Akt and p-Erk1/2 than control in organotypic slices (* *p* < 0.05, ** *p* < 0.01 vs. CRL, # *p* < 0.05 vs. 0.01; Figure 7A–C) whereas in mixed cells, we observed only a trend for *P. ginseng* treatment (Figure 8).

**Figure 9:**
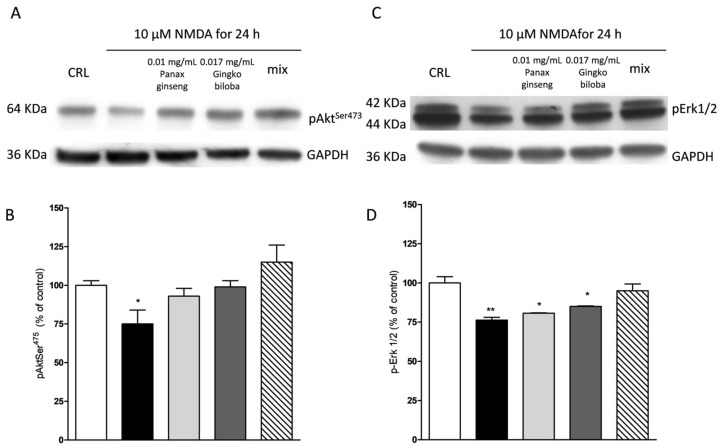
Effects of native *P. ginseng* and *G. biloba* GK501^®^ alone or in combination on MAP-kinase pathways in slices exposed to NMDA. Slices were exposed to the NMDA (10 µM for 24 h) and then processed for Western blot (WB). The extracts alone or as mix were presented to the incubation medium during NMDA exposure. (**A**,**C**) Representative blots for p-Akt or p-ERK1/2 or GADPH. Uncut original gels are available in the Supplementary File. (**B**,**D**) Quantitative analysis of immunoreactive bands, showing that the extracts alone are able to prevent a decrease in p-Akt induced by NMDA, whereas only the combination of the two extracts prevented the decrease of p-ERK1/2 phosphorylation induced by NMDA treatment. Bars represent the mean ± SEM of at least four experiments * *p* < 0.05 ** *p* < 0.01 vs. CRL (ANOVA + Tukey’s *w*-test).

**Figure 10:**
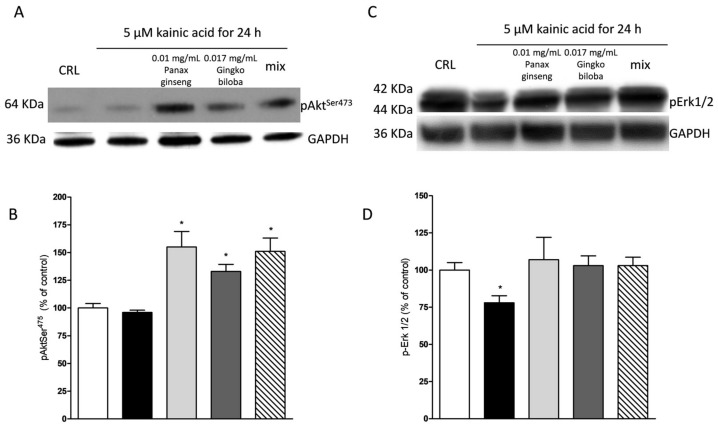
Effects of native *P. ginseng* and *G. biloba* GK501^®^ alone or in combination on MAP-kinase pathways in slices exposed to kainic acid. Slices were exposed to the kainic acid (5 µM for 24 h) and then processed for WB. The extracts alone or in combination were presented in the incubation medium during kainic acid exposure. (**A**,**C**) Representative blots for p-Akt or p-ERK1/2 or GADPH. Uncut original gels are available in the Supplementary File. (**B**,**D**) Quantitative analysis of immunoreactive bands, showing that the extract alone and in combination are able to induce an increased level of p-Akt in the presence of kainic acid and, furthermore, the two extracts prevented the decrease of p-ERK1/2 induced by kainic acid treatment. Bars represent the mean ± SEM of at least four experiments * *p* < 0.05 vs. CRL (ANOVA + Tukey’s *w*-test).

**Figure 11:**
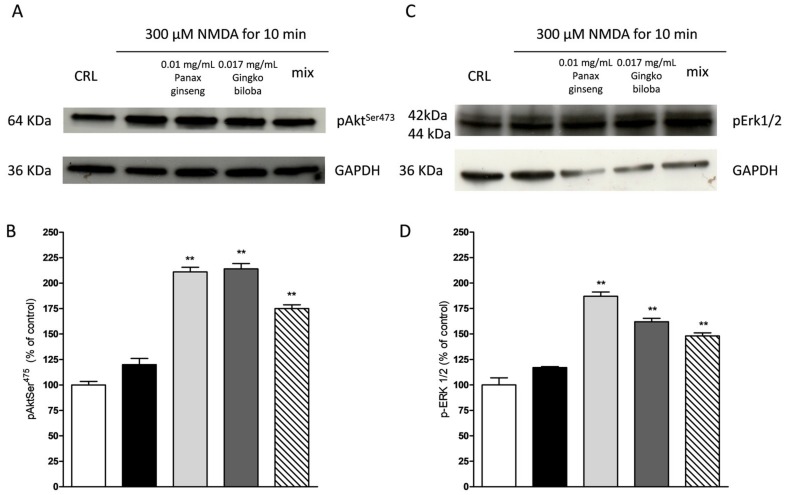
Effects of native *P. ginseng* and *G. biloba* GK501^®^ alone or in combination on MAP-kinase pathways in mixed cortical cells exposed to NMDA. Cells were exposed to 300 µM of NMDA for 10 min and then 24 h later were processed for WB. The extracts alone or in combination were presented in the incubation medium during NMDA exposure and after 24 h of recovery. (**A**,**C**) Representative blots for p-Akt or p-ERK1/2 or GADPH. Uncut original gels are available in the Supplementary File. (**B**,**D**) Quantitative analysis of immunoreactive bands, showing that the extracts alone and in combination are able to induce an increased level of p-Akt and of p-ERK1/2 in the presence of NMDA treatment. Bars represent the mean ± SEM of at least four experiments ** *p* < 0.01 vs. CRL (ANOVA + Tukey’s *w*-test).

NMDA induced a neuronal death in the CA1 subregion area of organotypic hippocampal slices and kainic acid in the CA3 region (Figure 2 and Figure 3); these effects were associated with a significant reduction of the hippocampal levels of p-Erk1/2 and p-Akt (Figure 9 and Figure 10). The extracts were able to revert partly the NMDA- and kainic acid-induced reduction of p-Akt and p-Erk1/2 reporting kinase levels (* *p* < 0.05 vs. CRL; Figure 9A–D and Figure 10A–D). In mixed cortical cells, NMDA per se did not change the levels of these proteins, whereas the incubation of NMDA with the single extracts or the mix induced a significant increase (** *p* < 0.01 vs. CRL; Figure 11). 

## 3. Discussion

Excitotoxicity-induced neurodegeneration is a determinant in various neurodegenerative diseases such as ischemia-induced brain damage, traumatic injury, disease, Parkinson’s disease, and Huntington’s disease [1]. Ginseng and ginkgo have been two of the most extensively used herbal medicines in Eastern Asian countries for more 2000 years [3,4,28]. Various studies have reported that ginseng and ginkgo protect against injury in numerous neurological diseases, including Parkinson’s disease [9,12], Alzheimer’s disease [14,16] and Huntington’s disease [17]. The neuroprotective effects were also observed for ginkgo in ischemia [18], against Aβ toxicity [19], and in Parkinson’s models [20,21]. Additionally, the activation of the PI3K/Akt pathways attenuates injury and alleviates damage to the brain in many neurological diseases [29], and ginseng and ginkgo have been shown to independently activate these pathways [13,30]. Neuroprotection of ginseng and ginkgo extracts is also related to their anti-inflammatory properties [31].

In this study, we utilized in vitro experimental models of excitotoxicity to explore the neuroprotective effect of native *P. ginseng* and *G. biloba* GK501^®^. We demonstrated in our models that both extracts reduced excitotoxic insult-induced neuronal damage in the hippocampus and cortex, and that the protective effects of the extracts were improved when used in combination. 

Brain function is dependent on good nutrition and a healthy lifestyle. Plant extracts such as *P. ginseng* and *G. biloba* GK501^®^ can also contribute to a healthy, cognitive function. In our study, we demonstrated that organotypic hippocampal slices and mixed cortical cells exposed to the extracts resulted in a marked attenuation of neuronal damage following excitotoxic injury. Slices exposed to the combination had reduced CA1 (NMDA model) cell death compared to slices treated with single extracts alone. When slices were treated with ginseng and ginkgo alone, a significant attenuation of neuronal damage induced by kainic acid in the CA3 area of organotypic slices was observed. Again, the combination of two extracts showed a more enhanced neuroprotective effect. The doses of extracts were selected in succession to achieve a probable clinical application, and they were extrapolated from other antecedent in vitro and in vivo preclinical studies and clinical use [26]. In the mixed cortical cells model of exitotoxicity, the neuroprotective effect of both ginseng and ginkgo was dose-dependent showing the greatest impact at the highest concentrations. As with the organotypic hippocampal slices model, treatment of the mixed cortical cells with the combination of extracts exhibited a higher degree of neuroprotection as compared to single extracts alone. When used at different time points after the NMDA insult, only the combination preserved significant neuroprotective effects. For the first time, our results provide evidence of the additive neuroprotective effects of the combination of *P. ginseng* and *G. biloba* GK501^®^ and their safety in neuron samples. Differentiating properties of the combination from the single extracts, our results suggest a role of the mix as a new potential approach in clinical settings to counteract those mechanisms that were found to be the cause of some neurodegenerative diseases such as Parkinson’s disease, Alzheimer’s disease, and Huntington’s disease. 

Furthermore, in this study we have demonstrated that native *P. ginseng* and *G. biloba* GK501^®^ extracts alone and in combination are able to activate pro-survival hippocampal signaling cascades including the Akt and the Erk1/2 pathways in organotypic hippocampal slices but not in mixed cortical cells. Activation of these pathways may, in part, increase the ability of neuronal cells to survive toxic insults such as the excitotoxic treatment that was mimicked by exposing hippocampal slices to NMDA and kainic acid. In this study, NMDA and kainic acid consistently induced death of neurons of the CA1 and CA3 regions of organotypic hippocampal slices, which correlated to the reduced hippocampal levels of p-Akt that we have previously observed [32] but also of p-Erk1/2. In the presence of the both extracts, NMDA- and kainate-induced cell death was reduced and, in parallel, the levels of p-Akt and p-Erk1/2 remained high in the hippocampus. In cortical cells, we did not observe a decrease of the protein levels after NMDA treatment, but in the presence of both extracts, the phosphorylation increased. This supports the idea that there is a causal relationship between the neuroprotection observed with *P. ginseng* G115^®^ and *G. biloba* GK501^®^ treatment and the activation of the PI3K/Akt and ERK1/2 pathways in the hippocampus. These findings are in agreement with a number of studies [3,10,13,33], suggesting that these extracts may be used as prevention or to improve the pharmacological treatment of neurological diseases. 

## 4. Materials and Methods 

### 4.1. Animals

Male and female Wistar rat pups (7–9 days old), mouse CD1 pups (1–2 days old), and pregnant CD1 mice were obtained from Charles River (MI, Italy). Animals were housed at 23 ± 1 °C under a 12 h light–dark cycle (lights on at 07:00) and were fed a standard laboratory diet with ad libitum access to water. Experiments and animal use procedures were in accordance with the National Institutes of Health Guide for the Care and Use of Laboratory Animals (NIH Publications No. 80-23, revised 1996). The experimental protocols were approved by the Animal Care Committee of the Department of Health Sciences, University of Florence, in compliance with the European Convention for the Protection of Vertebrate Animals used for Experimental and Other Scientific Purposes (ETS no. 123) project n 273/2016-PR (11/03/2016) and the European Communities Council Directive of 24 November 1986 (86/609/EEC). The authors further attest that all efforts were made to minimize the number of animals used and their suffering. 

### 4.2. Materials

Native extract of *P. ginseng* and *G. biloba* GK501^®^ extracts were a kind gift of Soho Flordis International Pty Ltd. Tissue culture reagents were obtained from Gibco-BRL (San Giuliano Milanese, Milano, Italy) and Sigma (St Louis, MO, USA).

### 4.3. Solubility Tests

The solubility tests were performed to determine the best solvent to use for each extract.

The *G. biloba* GK501^®^ and *P. ginseng* G115^®^ are contained in a commercially available product (Gincosan^®^ capsules) in the amounts of 60 mg and 100 mg per capsule.

According to Pharmacopoeia European, the standardized *P. ginseng* G115^®^ is obtained by ethanol extraction (40% *v*/ *v*) of the dried roots of *P. ginseng* and standardized on the total content of eight major ginsenosides (i.e., Rb1, Rb2, Rc, Rd, Re, Rf, Rg1, and Rg2) at 4.0% with a drug extract ratio (DER) of 3–7:1. 

The extract is in line with the monographs and Pharmacopoeias world-wide, including the European Pharmacopoeia, World Health Organization (WHO) Ginseng Monograph, and ESCOP (Scientific Foundation for Herbal Medicinal Products) Monographs.

The standardized *G. biloba* GK501^®^ is obtained by acetone/water extraction from the dried leaves of the *G. biloba*. The extract is standardized on the total content of ginkgo-flavone glycosides (22–27%) and of terpene lactones (5–7%). The extract conforms to the German Commission E Monograph and follows the indication of the WHO and ESCOP Monographs.

In our experiments, the native extracts of *P. ginseng,* which is the main constituent of the standardized *P. ginseng* G115^®^, and *G. biloba* GK501^®^ were appropriately solubilized to prepare standard solutions that reflected the composition of Gincosan^®^ and the posology of two capsules per day.

According to the specifications, 37.73 mg of this extract are equivalent to 100 mg of *P. ginseng* G115^®^. This aspect was considered for the solubility study and for the preparation of the samples for the pharmacological experiments.

Dimethyl sulfoxide (DMSO) and EtOH:H2O at different ratios (50:50, 60:40, and 70:30) were evaluated for suitability as solvents for both extracts. EtOH:H2O 50:50 was selected as the most appropriate solvent mixture, and was also compatible with the cell culture. The concentrations of standard solutions were: 1.08 mg/mL of native extract of *P. ginseng*, 1.78 mg/mL of *G. biloba* GK501^®^, and 2.79 mg/mL of mix.

### 4.4. Organotypic Rat Hippocampal Slice Models of Excitotoxicity

Organotypic hippocampal slice cultures were prepared as previously reported [34]. Briefly, hippocampi were removed from the brains of 7–9 days old Wistar rats (Charles River Laboratories, Milano, Italy), and transverse slices (420 µm) were prepared using a McIlwain tissue chopper and transferred onto semiporous membrane inserts and maintained in culture for 14 days in vitro. The slices were incubated with 5 µM kainic acid or 10 µM NMDA for 24 h [35,36]. Native *P. ginseng* and *G. biloba* GK501^®^ extracts alone or in combination (mix) were incubated during the 24 h treatment. Cell death was evaluated by using the fluorescent dye propidium iodide (5 µg/mL), and fluorescence was viewed using an inverted fluorescence microscope. Images were analyzed using morphometric analysis software. For cellular death, the CA3 and CA1 hippocampal subfields were identified, respectively, for kainic acid and NMDA toxicity, and were quantified using the image software v. 1.40g (ImageJ; NIH, Bethesda, MD, USA) that detected the optical density of PI fluorescence. 

### 4.5. Cortical Mice Cell Cultures Model of Excitotoxicity

Cortical mice cell cultures were prepared as previously reported [37]. Cerebral cortices were dissected from embryonic days (ED) 17–18 CD1 mice (Charles River, MI, Italy), cells were suspended and plated on a layer of confluent astrocytes. After 4–5 days in vitro (DIV), non-neuronal cell division was halted with cytosine arabinoside. After two weeks of maturation, the cells were exposed to 300 µM NMDA for 10 min as previously described [38,39]. NMDA neurotoxicity was obtained by exposing cultures to 300 µM NMDA for 10 min in HEPES medium after the original treatment medium was restored, and cells were stored in normal condition for 24 h, followed by assessment of the extent of neuronal death. To obtain the maximal neuronal injury, the cultures were exposed for 24 h to 1 mM glutamate. Native *P. ginseng* and *G. biloba* GK501^®^ extracts alone or as a mix were present in the incubation medium during NMDA treatment and during the 24 h of recovery period. The quantity of lactate dehydrogenase (LDH) released was measured 24 h after exposure to NMDA to evaluate the cell damage. Background LDH release was determined in control cultures. The resulting values correlated linearly with the degree of cell loss estimated by observation of cultures under phase-contrast microscopy. 

### 4.6. Western Blotting

Western blotting was conducted as previously described [27,37]. Four slices for sample and mixed cortical cells (3 wells/sample) were dissolved in 1% SDS. BCA (bicinchoninic acid) Protein Assays were used to quantify the total protein levels. Lysates (20 µg/lane of protein) were resolved by electrophoresis on a 4–20% SDS-polyacrylamide gels (Bio-Rad Laboratories, Hercules, CA, USA) and transferred onto nitrocellulose membranes. After blocking, the blots were incubated overnight at 4 °C with polyclonal-rabbit antibody against phospho-ERK1/2 (Thr202/Thr204) and phospho-Akt (Ser473) (all from Cell Signaling Technology, Beverly, MA, USA) diluted 1:1000 in TBS-T containing 5% bovine serum albumin. GAPDH was used as a loading control (monoclonal antibody purchased from Sigma (St Louis, MO, USA)). Immunodetection was performed with HRP-conjugated secondary antibodies (1:2000 anti-mouse, anti-rabbit, or anti-goat IgG from donkey, Amersham Biosciences, UK) in TBS-T containing 5% non-fat dry milk. After washing, membranes and reactive bands were detected using chemiluminescence (ECLplus; Euroclone, Padova, Italy). Quantity One analysis software was used for quantitative analysis (Bio-Rad, Hercules, CA, USA). Results are presented as the mean minus the standard error of the mean (SEM) of different gels and expressed as arbitrary unit (AU), which depicts the ratio between levels of target protein expression (for pERK1/2 both the bands were quantitated) and GAPDH normalized to basal levels [27,37]. 

### 4.7. Statistical Analysis

Data are presented as means ± SEM of n experiments. The statistical significance of differences between PI fluorescence intensities, LDH release, or Western blot optical densities was analyzed using one-way ANOVA with a post hoc Tukey’s *w*-test for multiple comparisons. All statistical calculations were performed using GRAPH-PAD PRISM v. 5 for Windows (GraphPad Software, San Diego, CA, USA). A probability value *p* < 0.05 was considered significant. 

## 5. Conclusions

*P. ginseng* and *G. biloba* extracts have a well-documented safety profile and have been demonstrated to be well tolerated for the treatment of moderate-to-severe disease. Preclinical studies using in vitro and in vivo models of neurological diseases and clinical trials have provided useful insights into the application of *P. ginseng* G115^®^ and *G. biloba* GK501^®^ as novel and natural therapeutic interventions for the treatment of brain diseases. These findings support the idea that the combination of neuroprotective strategies may become a valid and safe therapeutic intervention in the treatment of neurological disorders.

## Supplementary Materials

The following are available online at https://www.mdpi.com/1422-0067/20/23/5872/s1.

## Abbreviations

NMDA	*N*-methyl-d-aspartate
AMPA	α-amino-3-hydroxy-5-methyl-4-isoxazolepropionic acid;
PI	propidium iodide
ERK	extracellular-signal-regulated kinases
Akt	protein kinase B
mix	combination of *Panax ginseng* G115^®^ and *Ginkgo biloba* GK501^®^
LDH	lactate dehydrogenase
CNS	central nervous system
