# Supplementary material for: Enhanced Neuroprotective Effects of Panax ginseng G115® and Ginkgo biloba GK501® Combinations In Vitro Models of Excitotoxicity
Source: Int J Mol Sci. 2019 Nov 22;20(23):5872. doi: 10.3390/ijms20235872 (PMC6929202; doi:10.3390/ijms20235872)
Supplement: Supplementary file 1 [file ijms-20-05872-s001.pdf]

International Journal of *Molecular Sciences*

Original Western Blotting

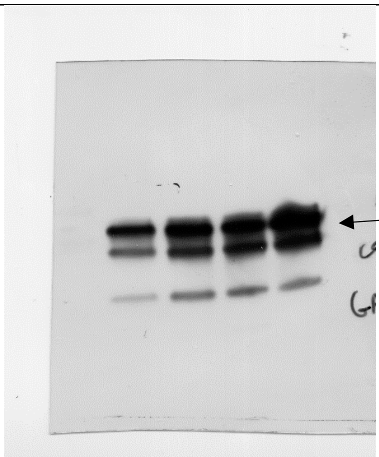

(a) p-ERK (42-44kDa)

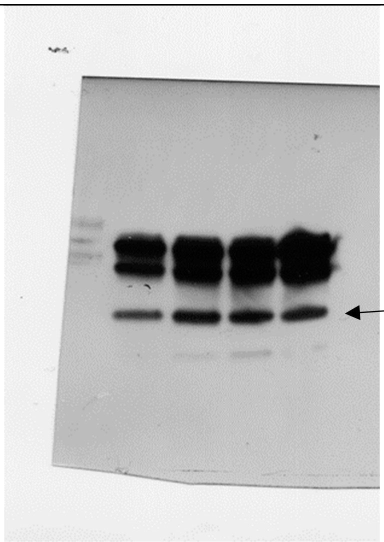

(b) GAPDHH (36kDa)

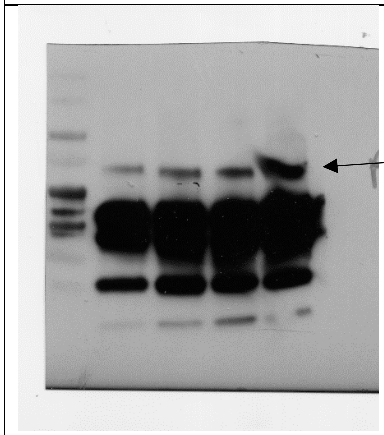

(c) p-AKT (64kDa)

Original western blot 1 (related to Fig 7). a-b: Representative immunoblotting of p-ERK1/2 and GAPDH and in (c) of p-AKT detected in protein extracts of rat organotypic hippocampal slices. Images belong to a single immunoblot whose exposure time varied to optimize protein-antibody combination

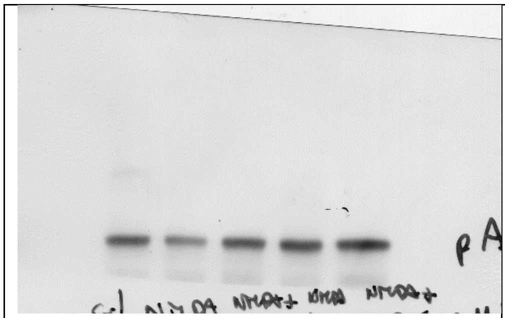

(a) p-AKT (64kDa)

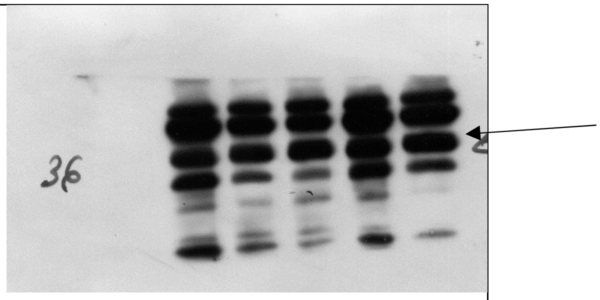

(b) GAPDH (36kDa)

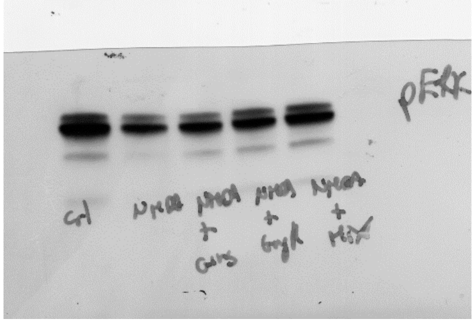

(c) p-ERK (42-44kDa)

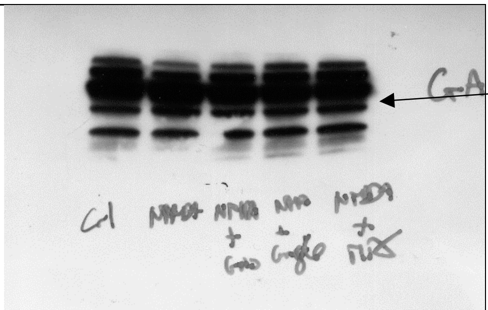

(d) GADPH (36kDa)

Original western blot 2 (related to Fig. 9A and Fig 9C). Representative immunoblotting p-AKT **(a)**, and GADPH (**b)** for Fig. 9A. Representative immunoblotting p-ERK1/2. **(c)** and GAPDH **(d)** for Fig 9C detected in protein extracts of of rat organotypic hippocampal slices. Image belongs to a single immunoblot whose exposure time varied to optimize protein-antibody combination.

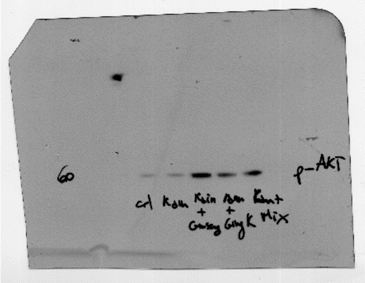

(a) p-AKT (64kDa)

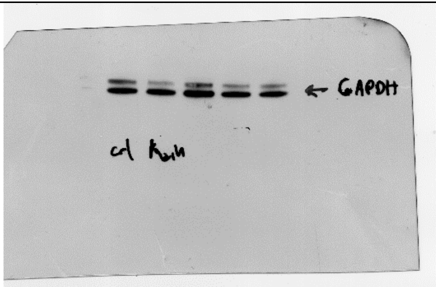

(b) GADPH (36kDa)

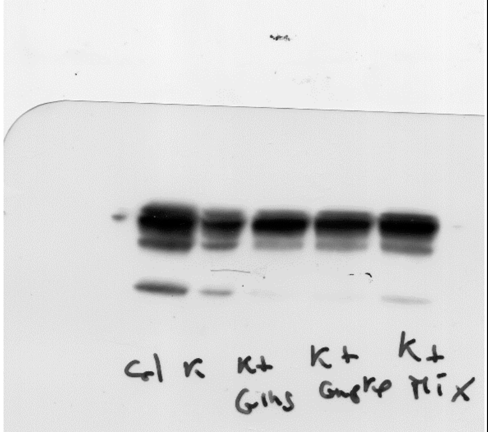

(c) p-ERK (42-44kDa)

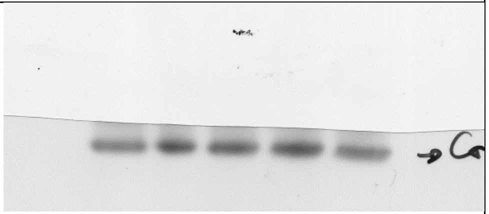

(d) GADPH (36kDa)

Original western blot 3 (related to Fig 10A and Fig 10 C). Representative immunoblotting p-AKT **(a)**, and GADPH (**b)** for Fig. 10 A. Representative immunoblotting p-ERK1/2. **(c)** and GAPDH **(d)** for Fig 10 B detected in protein extracts of of rat organotypic hippocampal slices Images belong to a single immunoblot whose exposure time varied to optimize protein-antibody combination.

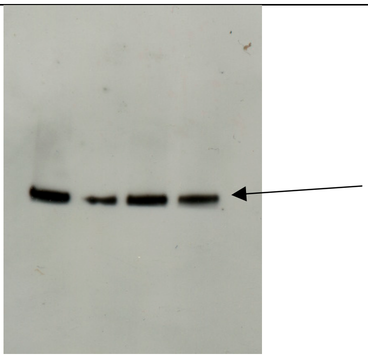

(a) p-AKT (64kDa)

(b) GAPDH (36kDa)

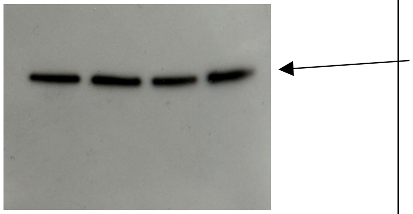

(c) p-ERK (42-44kDa)

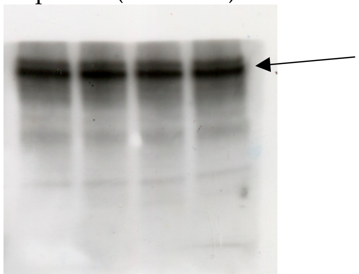

(d) GAPDH (36kDa)

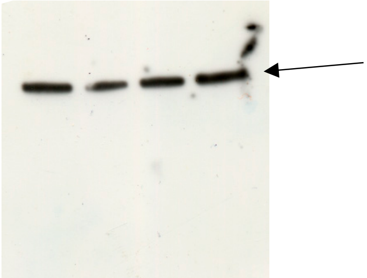

Original western blot 4 (related to Fig. 8A and Fig 8C). Representative immunoblotting p-AKT **(a)**, and GADPH (**b**) for Fig. 8A. Representative immunoblotting p-ERK1/2. **(c)** and GAPDH (**d**) for Fig 8C detected in protein extracts of mixed cortical cells. Image belongs to a single immunoblot whose exposure time varied to optimize protein-antibody combination.

(a) p-AKT (64kDa)

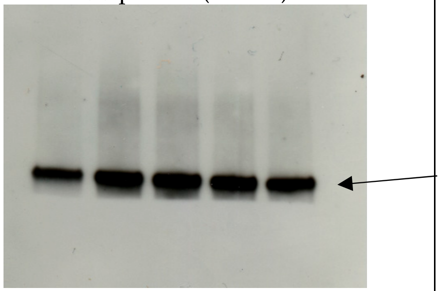

(b) GAPDH (36kDa)

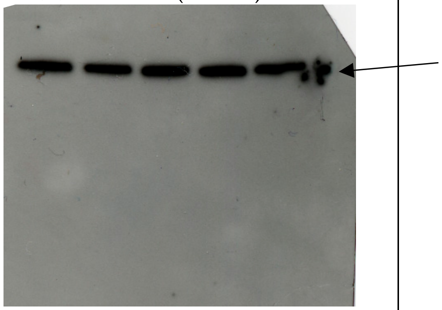

(c) p-ERK (42-44kDa) **(d)** GAPDH (36kDa)

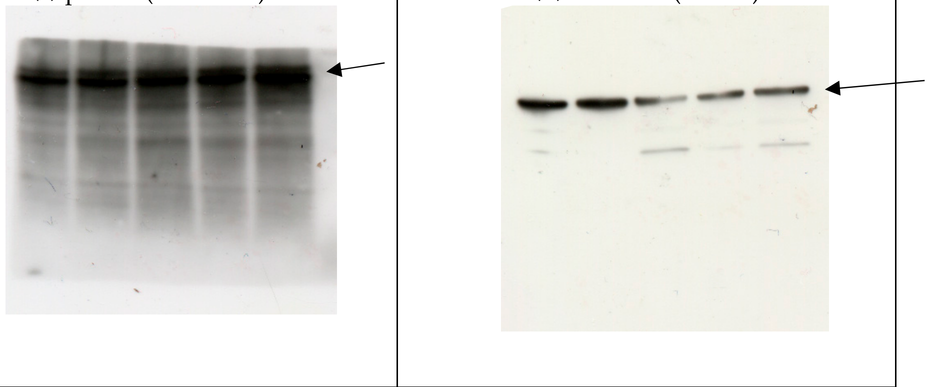

Original western blot 5 (related to Fig. 11 A and Fig 11 C). Representative immunoblotting p-AKT **(a)**, and GADPH (**b)** for Fig. 11A. Representative immunoblotting p-ERK1/2. **(c)** and GAPDH (**d**) for Fig 11C detected in protein extracts of mixed cortical cells exposed to NMDA. Image belongs to a single immunoblot whose exposure time varied to optimize protein-antibody combination.
